# Synthesis and crystal structure of di­aqua­(1,4,8,11-tetra­aza­cyclo­tetra­deca­ne)zinc(II) bis­(hydrogen 4-phospho­natobiphenyl-4′-carboxyl­ato)(1,4,8,11-tetra­aza­cyclo­tetra­deca­ne)zinc(II)

**DOI:** 10.1107/S2056989022004534

**Published:** 2022-05-17

**Authors:** Liudmyla V. Tsymbal, Irina L. Andriichuk, Vasile Lozan, Sergiu Shova, Yaroslaw D. Lampeka

**Affiliations:** a L. V. Pisarzhevskii Institute of Physical Chemistry of the National Academy of Sciences of Ukraine, Prospekt Nauki 31, Kiev 03028, Ukraine; b Institute of Chemistry of MES, Academiei str. 3, Chisinau 2028, Republic of Moldova; c ‘Petru Poni’ Institute of Macromolecular Chemistry, Department of Inorganic Polymers, Aleea Grigore Ghica Voda 41A, RO-700487 Iasi, Romania; University of Aberdeen, United Kingdom

**Keywords:** crystal structure, cyclam, zinc, 4-phospho­natobiphenyl-4′-carboxyl­ate, hydrogen bonding

## Abstract

In the asymmetric unit of the title compound, *trans*-di­aqua­(1,4,8,11-tetra­aza­cyclo­tetra­decane-κ^4^ *N* ^1^,*N* ^4^,*N* ^8^,*N* ^11^)zinc(II) *trans*-bis­(hydrogen 4-phospho­natobiphenyl-4′-carboxyl­ato-κ*O*)(1,4,8,11-tetra­aza­cyclo­tetra­decane-κ^4^ *N* ^1^,*N* ^4^,*N* ^8^,*N* ^11^)zinc(II), [Zn(C_10_H_24_N_4_)(H_2_O)_2_][Zn(C_13_H_9_O_5_P)_2_(C_10_H_24_N_4_)], both Zn atoms lie on crystallographic inversion centres and the atoms of the macrocycle in the cation are disordered over two sets of sites. In both macrocyclic units, the metal ions possess a tetra­gonally elongated ZnN_4_O_2_ octa­hedral environment formed by the four secondary N atoms of the macrocyclic ligand in the equatorial plane and the two *trans* O atoms of the water mol­ecules or anions in the axial positions, with the macrocyclic ligands adopting the most energetically favourable *trans*-III conformation. The average Zn—N bond lengths in both macrocyclic units do not differ significantly [2.112 (12) Å for the anion and 2.101 (3) Å for the cation] and are shorter than the average axial Zn—O bond lengths [2.189 (4) Å for phospho­nate and 2.295 (4) Å for aqua ligands]. In the crystal, the complex cations and anions are connected *via* hydrogen-bonding inter­actions between the N—H groups of the macrocycles, the O—H groups of coordinated water mol­ecules and the P—O—H groups of the acids as proton donors, and the O atoms of the phospho­nate and carboxyl­ate groups as acceptors, resulting in the formation of layers lying parallel to the (110) plane.

## Abstract 2

The coordination polyhedra of the zinc(II) ions in the complex cation and the anion of the title compound, *viz. trans*-ZnN_4_O_2_, are distorted octa­hedra. In the crystal, the hydrogen-bonding inter­actions between the N—H groups of the tetra­amine, the acidic groups of the anion and coordinated water mol­ecules result in formation of one-dimensional tapes running along the [1 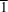 0] direction, which are further arranged in sheets lying parallel to the (001) plane.

## 1. Chemical context

Metal–organic frameworks (MOFs) – crystalline coordination polymers with permanent porosity – attract much current attention due to the possibilities of their applications in different areas, including gas storage, separation, sensing, catalysis, *etc*. (MacGillivray & Lukehart, 2014 ▸; Kaskel, 2016 ▸). Metal complexes of the tetra­aza-macrocycles, in particular cyclam (cyclam = 1,4,8,11-tetra­aza­cyclo­tetra­decane, C_10_H_24_N_4_, *L*), possessing high thermodynamic stability and kinetic inertness (Yatsimirskii & Lampeka, 1985 ▸), are popular metal-containing building units for the construction of MOFs (Lampeka & Tsymbal, 2004 ▸; Suh & Moon, 2007 ▸; Suh *et al.*, 2012 ▸; Stackhouse & Ma, 2018 ▸). The overwhelming majority of these materials are built up using oligo­carboxyl­ates as the bridging units (Rao *et al.*, 2004 ▸), though linkers with other coordinating groups, in particular oligo­phospho­nates, are also used for the construction of MOFs (Gagnon *et al.*, 2012 ▸). At the same time, hybrid bridging mol­ecules containing both phospho­nate and carboxyl­ate functional groups have been studied to a much lesser extent (see, for example, Heering *et al.*, 2016*b* ▸), though one can expect that the combination of different acidic donor groups in one ligand mol­ecule could open new possibilities for the creation of MOFs with specific chemical and structural features different from those inherent for MOFs formed by pure ligand classes. 
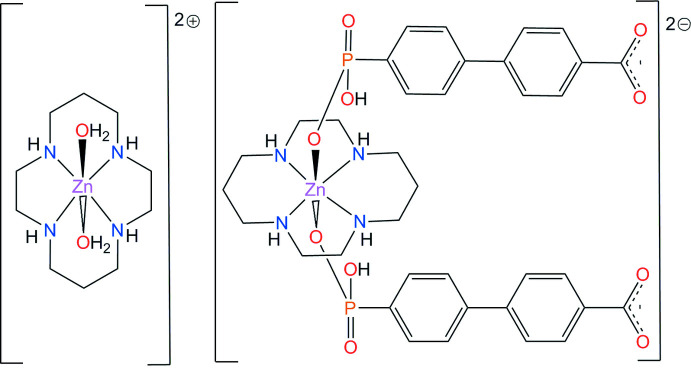


We report here the synthesis and crystal structure of the product of the reaction of [Zn(*L*)](ClO_4_)_2_ with 4-phos­phonato­biphenyl-4′-carb­oxy­lic acid (H_3_A) – the closest structural analogue of the ligand 4,4′-di­phenyldi­carboxyl­ate that is actively used for the preparation of different MOFs – namely, *trans*-di­aqua­(1,4,8,11-tetra­aza­cyclo­tetra­decane-κ^4^ *N* ^1^,*N* ^4^,*N* ^8^,*N* ^11^)zinc(II) *trans*-bis­(hydrogen 4-phospho­nato­bi­phenyl-4′-car­box­yl­ato-κ*O*)(1,4,8,11-tetra­aza­cyclo­tetra­decane-κ^4^ *N* ^1^,*N* ^4^,*N* ^8^,*N* ^11^)zinc(II), [Zn(*L*)(H_2_O)_2_][Zn(*L*)(HA)_2_], **I**. Though several ionic compounds and coordination polymers with this ligand have been reported (Heering *et al.*, 2016*a* ▸,*b* ▸), none of its complexes with macrocyclic cations have been described up to now.

## 2. Structural commentary

**Figure 1:**
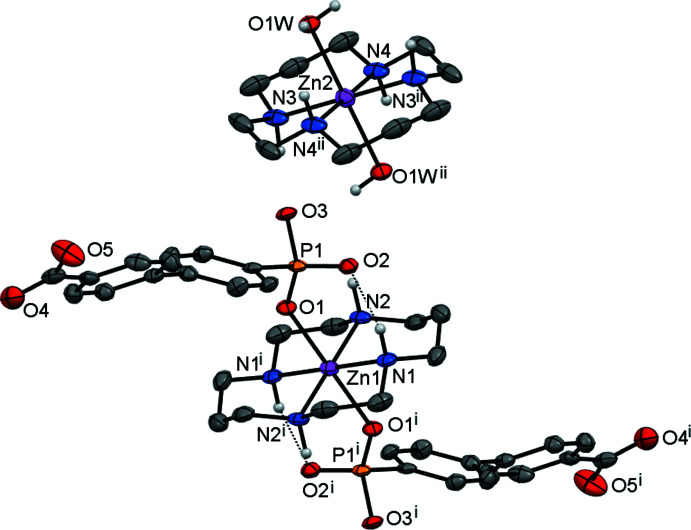
The extended asymmetric unit in **I**, showing the coordination environment of the Zn atoms and the atom-labelling scheme (displacement ellipsoids are drawn at the 30% probability level). C-bound H atoms have been omitted for clarity. Only one of two disordered components of the Zn2 cation is shown. Dotted lines represent intra-cation hydrogen-bonding inter­actions. [Symmetry codes: (i) −*x* + 2, −*y*, −*z* + 2; (ii) −*x* + 2, −*y*, −*z* + 1.]

The mol­ecular structure of the title compound, **I**, is shown in Fig. 1 ▸. Atom Zn1 (site symmetry 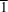 ) is coordinated by two monodentate doubly deprotonated acidic ligands HA^2−^ *via* their phospho­nate O-donor atoms, resulting in the formation of the [Zn1(*L*)(HA)_2_]^2−^ divalent anion, which is charge-balanced by the [Zn2(*L*)(H_2_O)_2_]^2+^ divalent cation (Zn2 site symmetry 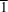 ). In the latter case, the macrocyclic ligand *L* is disordered over two orientations, with site occupancies of 50%, which are rotated around the O—Zn2—O axis by approximately 23°. The ligand *L* in both [Zn(*L*)] fragments adopts its energetically favoured *trans*-III conformation, with the five- and six-membered chelate rings in *gauche* and chair conformations, respectively (Bosnich *et al.*, 1965 ▸).

Both metal ions possess a tetra­gonally elongated *trans*-ZnN_4_O_2_ octa­hedral environment formed by the four secondary N atoms of the macrocyclic ligand in the equatorial plane and the two O atoms of the anions or water mol­ecules in the axial positions (Table 1 ▸). The location of the metal ions on inversion centres enforces strict planarity of the ZnN_4_ coordination moieties. The directivity of the axial Zn—O bonds is nearly orthogonal to the ZnN_4_ plane.

The average Zn—N bond lengths in both macrocyclic units do not differ significantly [2.112 (12) Å for Zn1 and 2.101 (3) Å for Zn2] and are shorter than the average axial Zn—O bond lengths. The Zn—O distance for the phos­pho­n­ate group [2.189 (4) Å] is shorter than that for the aqua ligand [2.295 (4) Å], reflecting the stronger donating properties of the anion. Thus, analogous to the situation for caboxylate groups coordinated to aza-macrocyclic cations (Tsymbal *et al.*, 2021 ▸), the Zn—O inter­actions are reinforced by intra­molecular hydrogen bonding between the secondary amino group (N1—H1) of ligand *L* and the O2 atom of the phospho­nate fragment (Table 2 ▸).

The benzene rings in the HA^2−^ anion in **I** are tilted with respect to each other [the angle between their mean planes is 40.4 (2)°], while the uncoordinated carboxyl­ate group is close to being coplanar with the corresponding aromatic fragment [12.3 (2)°]. This carboxyl­ate group displays a high degree of electronic delocalization [the C23—O4 and C23—O5 bond lengths are 1.251 (8) and 1.258 (8) Å, respectively], as does part of the coordinated phospho­nate group [1.503 (4) and 1.511 (4) Å for the P1—O1 and P1—O2 bond lengths, respectivey]. The protonated P—O3H bond [1.583 (4) Å] is not involved in delocalization.

## 3. Supra­molecular features

**Figure 2:**
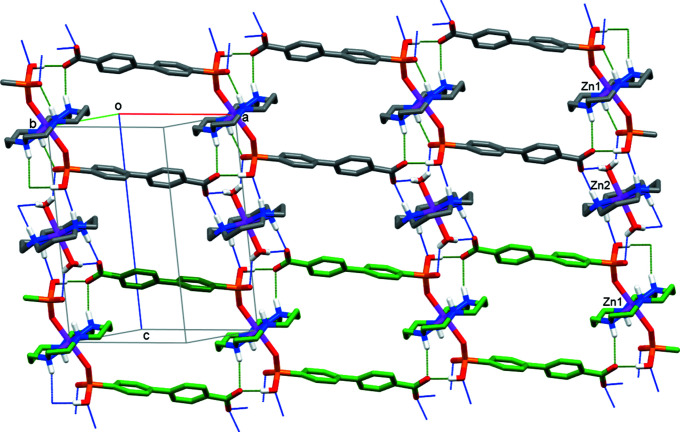
The hydrogen-bonded tape (C atoms in green) and sheet parallel to the (110) plane in **I**. H atoms at C atoms have been omitted, as has one disorder component of the macrocyclic Zn2 cation. Intra- and inter-tape hydrogen bonds are shown as dashed lines in green and blue, respectively; intra­molecular N1—H1⋯O2 hydrogen bonds are not depicted.

The crystals of **I** are composed of [Zn1(*L*)(HA)_2_]^2−^ anions and [Zn2(*L*)(H_2_O)_2_]^2+^ cations that are connected by numerous hydrogen bonds (Table 2 ▸). In particular, due to hydrogen bonding between the protonated phospho­nate P1—O3—H fragments and the secondary amino N2—H2 groups of the macrocycle *L* as proton donors, and carboxyl­ate atoms O4 [at (*x* + 1, *y* − 1, *z*)] as acceptors, the complex anions are arranged into one-dimensional tapes running along the [1 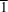 0] direction (Fig. 2 ▸). These tapes are further connected into two-dimensional arrays lying parallel to the (110) plane by virtue of O—H⋯O and N—H⋯O hydrogen bonding between the O1*W* coordinated water mol­ecule and the amino N3—H3 and N4—H4 groups as donors, and the phospho­nate and carboxyl­ate atoms O2 [at (−*x* + 2, −*y*, −*z* + 1)], O3 and O5 [at (*x* + 1, *y* − 1, *z*) and (−*x* + 1, −*y* + 1, −*z* + 1)] as acceptors (Fig. 2 ▸). The distances Zn1⋯Zn1(*x* + 1, *y* − 1, *z*) and Zn2⋯Zn2(*x* + 1, *y* − 1, *z*) in the [1 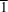 0] direction are 14.179 (2) Å, while the Zn1⋯Zn2 distance is 8.131 (1) Å. There are no hydrogen-bonding contacts between the layers and the three-dimensional coherence of the crystal is provided by van der Waals inter­actions.

## 4. Database survey

A search of the Cambridge Structural Database (CSD, Version 5.43, last update March 2022; Groom *et al.*, 2016 ▸) indicated that several ionic compounds including ammonium and hexa­amine cobalt(III) cations (refcodes SEDDUD and SEDFEP, respectively; Heering *et al.*, 2016*a* ▸) and coordination polymers formed by zinc(II) (UNISOB and UNISUH), cadmium(II) (UNITES) and mercury(II) ions (UNIWEV; Heering *et al.*, 2016*b* ▸) have been structurally characterized to date. In the polymeric complexes, the phospho­nate groups of the ligands display a μ_3_–μ_5_ bridging function and form two-dimensional metal–oxo layers. The complexation behaviour of the carboxyl­ate groups determines the dimensionality of the polymeric systems formed. If, like in **I**, they are not coordinated, the metal–oxo layers are simply decorated with ligand mol­ecules (UNISOB and UNIWEV). At the same time, the μ_2_- or μ_3_-bridging function of the carboxyl­ate groups results in the formation of another kind of metal–oxo layer, thus producing three-dimensional coordination polymers (UNISUH and UNITES), in which the ligand mol­ecules act as pillars. Inter­estingly, the tilting of the benzene rings in the ligand in polymeric complexes is much smaller that in **I** and does not exceed 7° (UNITES).

## 5. Synthesis and crystallization

All chemicals and solvents used in this work were purchased from Sigma–Aldrich and were used without further purification. The acid H_3_A was synthesized according to a procedure described previously (Heering *et al.*, 2016*b* ▸). The complex [Zn(*L*)](ClO_4_)_2_ was prepared by mixing equimolar amounts of *L* and zinc perchlorate hexa­hydrate in ethanol.

For the preparation of the title compound, **I**, a solution of [Zn(*L*)](ClO_4_)_2_ (23 mg, 0.06 mmol) in water (2 ml) was added to a di­methyl­formamide (DMF) solution (3 ml) of H_3_A (11 mg, 0,04 mmol) containing tri­ethyl­amine (0.05 ml). A white precipitate, which had formed over several days, was filtered off, washed with small amounts of dimethylformamide (DMF) and diethyl ether, and dried in air (yield: 6.7 mg, 15% based on the acid). Analysis calculated (%) for C_46_H_70_N_8_O_12_P_2_Zn_2_: C 49.34, H 6.30, N 10.01; found: C 49.45, H 6.41, N 10.21. Single crystals of **I** suitable for X-ray diffraction analysis were selected from the sample resulting from the synthesis. **Caution! Perchlorate salts of metal complexes are potentially explosive and should be handled with care.**

## 6. Refinement

Crystal data, data collection and structure refinement details are summarized in Table 3 ▸. The H atoms in **I** were placed in geometrically idealized positions and constrained to ride on their parent atoms, with C—H distances of 0.93 (ring H atoms) and 0.97 Å (methyl­ene H atoms), and N—H distances of 0.98 Å, with *U*_iso_(H) values of 1.2*U*_eq_ of the parent atoms.

## Supplementary Material

Crystal structure: contains datablock(s) I, global. DOI: 10.1107/S2056989022004534/hb8017sup1.cif

Structure factors: contains datablock(s) I. DOI: 10.1107/S2056989022004534/hb8017Isup2.hkl

CCDC reference: 2166638

Additional supporting information:  crystallographic information; 3D view; checkCIF report

## Figures and Tables

**Table 1:** Selected geometric parameters (Å, °)

Zn1—O1	2.189 (4)	Zn2—O1*W*	2.295 (4)
Zn1—N1	2.099 (5)	Zn2—N3	2.104 (7)
Zn1—N2	2.125 (4)	Zn2—N4	2.092 (7)
			
N1—Zn1—N2^i^	85.27 (19)	N4—Zn2—N3	96.8 (4)
N1—Zn1—N2	94.73 (18)	N4—Zn2—N3^ii^	83.2 (4)

Symmetry codes: (i) 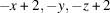 ; (ii) 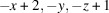 .

**Table 2:** Hydrogen-bond geometry (Å, °)

*D*—H⋯*A*	*D*—H	H⋯*A*	*D*⋯*A*	*D*—H⋯*A*
N1—H1⋯O2	0.98	1.98	2.923 (6)	161
N2—H2⋯O4^iii^	0.98	2.26	3.220 (6)	166
N3—H3⋯O3	0.98	2.11	3.076 (10)	168
N4—H4⋯O5^iii^	0.98	1.84	2.815 (11)	178
O3—H3*C*⋯O4^iii^	0.86	1.75	2.597 (6)	167
O1*W*—H1*WA*⋯O2^ii^	0.87	2.08	2.735 (5)	132
O1*W*—H1*WB*⋯O5^iv^	0.86	1.82	2.668 (6)	169

Symmetry codes: (ii) 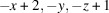 ; (iii) 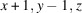 ; (iv) 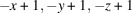 .

**Table 3:** Experimental details

Crystal data	
Chemical formula	[Zn(C_10_H_24_N_4_)(H_2_O)_2_][Zn(C_13_H_9_O_5_P)_2_(C_10_H_24_N_4_)]
*M*_r_	1119.78
Crystal system, space group	Triclinic, *P* 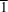
Temperature (K)	296
*a*, *b*, *c* (Å)	8.8781 (15), 9.3224 (14), 16.2627 (14)
α, β, γ (°)	102.759 (10), 90.777 (11), 102.315 (14)
*V* (Å^3^)	1279.9 (3)
*Z*	1
Radiation type	Mo *K*α
μ (mm^−1^)	1.07
Crystal size (mm)	0.3 × 0.1 × 0.05

Data collection	
Diffractometer	Rigaku Xcalibur Eos
Absorption correction	Multi-scan (*CrysAlis PRO*; Rigaku OD, 2019 ▸)
*T*_min_, *T*_max_	0.866, 1.000
No. of measured, independent and observed [*I* > 2σ(*I*)] reflections	9378, 4514, 3242
*R*_int_	0.081
(sin θ/λ)_max_ (Å^−1^)	0.595

Refinement	
*R*[*F* ^2^ > 2σ(*F* ^2^)], *wR*(*F* ^2^), *S*	0.080, 0.203, 1.06
No. of reflections	4514
No. of parameters	317
No. of restraints	41
H-atom treatment	H atoms treated by a mixture of independent and constrained refinement
Δρ_max_, Δρ_min_ (e Å^−3^)	0.91, −0.69

Computer programs: *CrysAlis PRO* (Rigaku OD, 2019 ▸), *SHELXT2018* (Sheldrick, 2015*a* ▸), *SHELXL2018* (Sheldrick, 2015*b* ▸), *Mercury* (Macrae *et al.*, 2020 ▸) and *publCIF* (Westrip, 2010 ▸).

## supplementary crystallographic information

### Crystal data

**Table:**

[Zn(C_10_H_24_N_4_)(H_2_O)_2_][Zn(C_13_H_9_O_5_P)_2_(C_10_H_24_N_4_)]	*Z* = 1
*M**_r_* = 1119.78	*F*(000) = 588
Triclinic, *P*1	*D*_x_ = 1.453 Mg m^−^^3^
*a* = 8.8781 (15) Å	Mo *K*α radiation, λ = 0.71073 Å
*b* = 9.3224 (14) Å	Cell parameters from 1677 reflections
*c* = 16.2627 (14) Å	θ = 2.3–25.6°
α = 102.759 (10)°	µ = 1.07 mm^−^^1^
β = 90.777 (11)°	*T* = 296 K
γ = 102.315 (14)°	Block, clear light colourless
*V* = 1279.9 (3) Å^3^	0.3 × 0.1 × 0.05 mm

### Data collection

**Table:**

Rigaku Xcalibur Eos diffractometer	4514 independent reflections
Radiation source: fine-focus sealed X-ray tube, Enhance (Mo) X-ray Source	3242 reflections with *I* > 2σ(*I*)
Graphite monochromator	*R*_int_ = 0.081
Detector resolution: 8.0797 pixels mm^-1^	θ_max_ = 25.0°, θ_min_ = 2.3°
ω scans	*h* = −10→10
Absorption correction: multi-scan (CrysAlis PRO; Rigaku OD, 2019)	*k* = −10→11
*T*_min_ = 0.866, *T*_max_ = 1.000	*l* = −19→19
9378 measured reflections	

### Refinement

**Table:**

Refinement on *F*^2^	Primary atom site location: dual
Least-squares matrix: full	Hydrogen site location: mixed
*R*[*F*^2^ > 2σ(*F*^2^)] = 0.080	H atoms treated by a mixture of independent and constrained refinement
*wR*(*F*^2^) = 0.203	*w* = 1/[σ^2^(*F*_o_^2^) + (0.084*P*)^2^ + 0.5432*P*] where *P* = (*F*_o_^2^ + 2*F*_c_^2^)/3
*S* = 1.06	(Δ/σ)_max_ < 0.001
4514 reflections	Δρ_max_ = 0.91 e Å^−^^3^
317 parameters	Δρ_min_ = −0.69 e Å^−^^3^
41 restraints	

### Special details

**Table:**

Geometry. All esds (except the esd in the dihedral angle between two l.s. planes) are estimated using the full covariance matrix. The cell esds are taken into account individually in the estimation of esds in distances, angles and torsion angles; correlations between esds in cell parameters are only used when they are defined by crystal symmetry. An approximate (isotropic) treatment of cell esds is used for estimating esds involving l.s. planes.

### Fractional atomic coordinates and isotropic or equivalent isotropic displacement parameters (Å^2^)

**Table:**

	*x*	*y*	*z*	*U*_iso_*/*U*_eq_	Occ. (<1)
Zn1	1.000000	0.000000	1.000000	0.0289 (3)	
P1	0.88329 (17)	0.02758 (16)	0.80359 (8)	0.0265 (4)	
O1	0.8559 (4)	−0.0163 (4)	0.8866 (2)	0.0323 (9)	
O2	1.0432 (4)	0.1165 (4)	0.7945 (2)	0.0321 (9)	
O3	0.8352 (5)	−0.1164 (4)	0.7280 (2)	0.0342 (10)	
H3C	0.909808	−0.161970	0.728072	0.06 (2)*	
O4	0.0291 (5)	0.7128 (5)	0.7344 (3)	0.0507 (12)	
O5	0.1942 (7)	0.7954 (6)	0.6441 (3)	0.0742 (17)	
N1	1.1789 (5)	0.1580 (5)	0.9652 (3)	0.0339 (12)	
H1	1.153015	0.161569	0.907023	0.041*	
N2	1.0824 (6)	−0.1907 (5)	0.9371 (3)	0.0331 (12)	
H2	1.051793	−0.211859	0.876801	0.040*	
C1	1.3309 (7)	0.1235 (8)	0.9670 (4)	0.0475 (17)	
H1A	1.364444	0.129596	1.024910	0.057*	
H1B	1.404031	0.198141	0.945943	0.057*	
C2	1.3313 (7)	−0.0338 (7)	0.9136 (4)	0.0472 (17)	
H2A	1.282632	−0.043165	0.858312	0.057*	
H2B	1.437937	−0.040350	0.905594	0.057*	
C3	1.2535 (7)	−0.1652 (7)	0.9470 (4)	0.0453 (17)	
H3A	1.285584	−0.254901	0.917223	0.054*	
H3B	1.285174	−0.147785	1.006389	0.054*	
C4	0.9991 (8)	−0.3146 (7)	0.9716 (4)	0.0480 (18)	
H4A	1.046006	−0.309242	1.026683	0.058*	
H4B	1.005633	−0.410042	0.934768	0.058*	
C5	1.1696 (8)	0.3063 (7)	1.0205 (4)	0.0474 (17)	
H5A	1.223716	0.387661	0.996402	0.057*	
H5B	1.218427	0.317228	1.075963	0.057*	
C11	0.7481 (7)	0.1412 (6)	0.7863 (3)	0.0273 (13)	
C12	0.5919 (7)	0.0848 (6)	0.7650 (3)	0.0330 (14)	
H12	0.550559	−0.017794	0.758556	0.040*	
C13	0.4965 (7)	0.1788 (6)	0.7532 (4)	0.0350 (14)	
H13	0.391696	0.138655	0.739331	0.042*	
C14	0.5548 (7)	0.3329 (6)	0.7618 (3)	0.0317 (14)	
C15	0.7119 (7)	0.3890 (6)	0.7822 (4)	0.0358 (15)	
H15	0.753957	0.491104	0.787393	0.043*	
C16	0.8059 (7)	0.2947 (6)	0.7949 (3)	0.0331 (14)	
H16	0.910507	0.334874	0.809487	0.040*	
C17	0.4534 (7)	0.4353 (6)	0.7474 (3)	0.0294 (13)	
C18	0.3040 (7)	0.4188 (6)	0.7760 (3)	0.0345 (14)	
H18	0.266975	0.343425	0.804391	0.041*	
C19	0.2111 (7)	0.5140 (6)	0.7622 (4)	0.0373 (15)	
H19	0.113715	0.503674	0.783539	0.045*	
C20	0.2585 (7)	0.6225 (6)	0.7183 (3)	0.0310 (14)	
C21	0.4062 (8)	0.6399 (7)	0.6901 (4)	0.0424 (17)	
H21	0.441059	0.713837	0.660376	0.051*	
C22	0.5029 (7)	0.5488 (7)	0.7054 (4)	0.0410 (16)	
H22	0.602640	0.564311	0.687123	0.049*	
C23	0.1516 (8)	0.7174 (7)	0.6970 (4)	0.0397 (16)	
Zn2	1.000000	0.000000	0.500000	0.0363 (3)	
O1W	0.8744 (5)	−0.0550 (4)	0.3688 (2)	0.0394 (10)	
H1WA	0.950153	−0.050667	0.335769	0.059*	
H1WB	0.860423	0.028953	0.359289	0.059*	
N3	0.7873 (9)	−0.0897 (10)	0.5448 (6)	0.0419 (15)	0.5
H3	0.805715	−0.081913	0.605351	0.050*	0.5
N4	1.0871 (12)	−0.1955 (9)	0.4820 (6)	0.0419 (15)	0.5
H4	1.123687	−0.201773	0.537935	0.050*	0.5
C6	0.7127 (18)	−0.2479 (14)	0.5069 (10)	0.063 (2)	0.5
H6A	0.664219	−0.253928	0.452095	0.075*	0.5
H6B	0.632279	−0.283288	0.542095	0.075*	0.5
C7	0.8282 (15)	−0.3491 (16)	0.4932 (9)	0.063 (2)	0.5
H7A	0.862152	−0.349203	0.550088	0.075*	0.5
H7B	0.766981	−0.448083	0.466108	0.075*	0.5
C8	0.9767 (16)	−0.3350 (14)	0.4479 (8)	0.063 (2)	0.5
H8A	1.023645	−0.419755	0.448358	0.075*	0.5
H8B	0.949025	−0.338235	0.389488	0.075*	0.5
C9	1.2244 (19)	−0.165 (2)	0.4347 (15)	0.059 (3)	0.5
H9A	1.192948	−0.182814	0.375229	0.070*	0.5
H9B	1.289179	−0.233766	0.440579	0.070*	0.5
C10	0.6842 (17)	0.0081 (17)	0.5368 (9)	0.059 (3)	0.5
H10A	0.646497	−0.009429	0.478382	0.070*	0.5
H10B	0.597397	−0.008959	0.571702	0.070*	0.5
N3X	0.7879 (9)	−0.0031 (11)	0.5569 (6)	0.0419 (15)	0.5
H3X	0.808176	−0.003809	0.616184	0.050*	0.5
N4X	0.9879 (11)	−0.2316 (8)	0.4855 (6)	0.0419 (15)	0.5
H4X	1.020262	−0.249725	0.539454	0.050*	0.5
C6X	0.6707 (17)	−0.1419 (13)	0.5207 (9)	0.063 (2)	0.5
H6XA	0.646379	−0.143247	0.462195	0.075*	0.5
H6XB	0.577179	−0.137626	0.550435	0.075*	0.5
C7X	0.7152 (18)	−0.2923 (15)	0.5230 (10)	0.063 (2)	0.5
H7XA	0.758274	−0.287024	0.578983	0.075*	0.5
H7XB	0.623653	−0.373619	0.510803	0.075*	0.5
C8X	0.8334 (15)	−0.3249 (17)	0.4581 (9)	0.063 (2)	0.5
H8XA	0.838150	−0.430111	0.449835	0.075*	0.5
H8XB	0.799859	−0.308891	0.404535	0.075*	0.5
C9X	1.1027 (16)	−0.2646 (19)	0.4236 (8)	0.059 (3)	0.5
H9XA	1.060250	−0.270607	0.367405	0.070*	0.5
H9XB	1.124940	−0.361687	0.424735	0.070*	0.5
C10X	0.743 (2)	0.1386 (19)	0.5572 (16)	0.059 (3)	0.5
H10C	0.670054	0.158493	0.599963	0.070*	0.5
H10D	0.695124	0.135023	0.502503	0.070*	0.5

### Atomic displacement parameters (Å^2^)

**Table:**

	*U*^11^	*U*^22^	*U*^33^	*U*^12^	*U*^13^	*U*^23^
Zn1	0.0359 (6)	0.0307 (5)	0.0213 (5)	0.0112 (4)	−0.0001 (4)	0.0050 (4)
P1	0.0354 (9)	0.0329 (8)	0.0130 (7)	0.0148 (7)	−0.0034 (6)	0.0024 (6)
O1	0.038 (2)	0.042 (2)	0.019 (2)	0.0099 (19)	−0.0004 (17)	0.0121 (17)
O2	0.036 (2)	0.041 (2)	0.021 (2)	0.0126 (19)	−0.0033 (17)	0.0062 (17)
O3	0.042 (3)	0.039 (2)	0.021 (2)	0.017 (2)	−0.0097 (18)	−0.0023 (17)
O4	0.059 (3)	0.051 (3)	0.050 (3)	0.033 (2)	−0.003 (2)	0.007 (2)
O5	0.111 (5)	0.099 (4)	0.052 (3)	0.078 (4)	0.023 (3)	0.046 (3)
N1	0.037 (3)	0.045 (3)	0.017 (2)	0.007 (2)	−0.005 (2)	0.007 (2)
N2	0.048 (3)	0.039 (3)	0.015 (2)	0.020 (2)	−0.001 (2)	0.003 (2)
C1	0.033 (4)	0.071 (5)	0.035 (4)	0.001 (3)	−0.007 (3)	0.016 (3)
C2	0.041 (4)	0.073 (5)	0.032 (4)	0.027 (4)	0.000 (3)	0.007 (3)
C3	0.051 (4)	0.060 (4)	0.025 (3)	0.026 (4)	−0.011 (3)	−0.002 (3)
C4	0.073 (5)	0.039 (4)	0.030 (3)	0.018 (4)	−0.006 (3)	0.000 (3)
C5	0.061 (5)	0.041 (4)	0.034 (4)	0.000 (3)	−0.010 (3)	0.006 (3)
C11	0.040 (4)	0.032 (3)	0.012 (3)	0.019 (3)	−0.003 (2)	−0.001 (2)
C12	0.040 (4)	0.030 (3)	0.029 (3)	0.011 (3)	−0.003 (3)	0.004 (3)
C13	0.030 (3)	0.033 (3)	0.038 (3)	0.003 (3)	−0.002 (3)	0.006 (3)
C14	0.038 (4)	0.038 (3)	0.023 (3)	0.015 (3)	0.003 (3)	0.009 (3)
C15	0.045 (4)	0.025 (3)	0.039 (4)	0.013 (3)	−0.005 (3)	0.006 (3)
C16	0.033 (3)	0.037 (3)	0.026 (3)	0.008 (3)	−0.008 (3)	0.000 (3)
C17	0.038 (3)	0.030 (3)	0.021 (3)	0.010 (3)	−0.004 (2)	0.004 (2)
C18	0.039 (4)	0.037 (3)	0.030 (3)	0.013 (3)	0.002 (3)	0.009 (3)
C19	0.037 (4)	0.043 (4)	0.036 (3)	0.016 (3)	0.002 (3)	0.009 (3)
C20	0.040 (4)	0.033 (3)	0.022 (3)	0.022 (3)	−0.001 (3)	−0.002 (2)
C21	0.069 (5)	0.041 (4)	0.026 (3)	0.025 (3)	0.003 (3)	0.012 (3)
C22	0.043 (4)	0.046 (4)	0.041 (4)	0.020 (3)	0.009 (3)	0.013 (3)
C23	0.057 (4)	0.038 (4)	0.026 (3)	0.027 (3)	−0.010 (3)	−0.003 (3)
Zn2	0.0392 (6)	0.0365 (6)	0.0346 (6)	0.0141 (5)	0.0030 (5)	0.0053 (4)
O1W	0.044 (3)	0.053 (3)	0.024 (2)	0.012 (2)	−0.0033 (19)	0.0136 (19)
N3	0.064 (4)	0.046 (4)	0.022 (2)	0.023 (4)	−0.001 (3)	0.011 (3)
N4	0.064 (4)	0.046 (4)	0.022 (2)	0.023 (4)	−0.001 (3)	0.011 (3)
C6	0.081 (5)	0.051 (4)	0.040 (4)	−0.018 (4)	−0.018 (3)	0.011 (3)
C7	0.081 (5)	0.051 (4)	0.040 (4)	−0.018 (4)	−0.018 (3)	0.011 (3)
C8	0.081 (5)	0.051 (4)	0.040 (4)	−0.018 (4)	−0.018 (3)	0.011 (3)
C9	0.068 (6)	0.096 (7)	0.030 (4)	0.054 (5)	0.017 (5)	0.019 (5)
C10	0.068 (6)	0.096 (7)	0.030 (4)	0.054 (5)	0.017 (5)	0.019 (5)
N3X	0.064 (4)	0.046 (4)	0.022 (2)	0.023 (4)	−0.001 (3)	0.011 (3)
N4X	0.064 (4)	0.046 (4)	0.022 (2)	0.023 (4)	−0.001 (3)	0.011 (3)
C6X	0.081 (5)	0.051 (4)	0.040 (4)	−0.018 (4)	−0.018 (3)	0.011 (3)
C7X	0.081 (5)	0.051 (4)	0.040 (4)	−0.018 (4)	−0.018 (3)	0.011 (3)
C8X	0.081 (5)	0.051 (4)	0.040 (4)	−0.018 (4)	−0.018 (3)	0.011 (3)
C9X	0.068 (6)	0.096 (7)	0.030 (4)	0.054 (5)	0.017 (5)	0.019 (5)
C10X	0.068 (6)	0.096 (7)	0.030 (4)	0.054 (5)	0.017 (5)	0.019 (5)

### Geometric parameters (Å, º)

**Table:**

Zn1—O1^i^	2.189 (4)	C21—C22	1.385 (8)
Zn1—O1	2.189 (4)	C22—H22	0.9300
Zn1—N1^i^	2.099 (5)	Zn2—O1W^ii^	2.295 (4)
Zn1—N1	2.099 (5)	Zn2—O1W	2.295 (4)
Zn1—N2^i^	2.125 (4)	Zn2—N3	2.104 (7)
Zn1—N2	2.125 (4)	Zn2—N3^ii^	2.104 (7)
P1—O1	1.503 (4)	Zn2—N4^ii^	2.092 (7)
P1—O2	1.511 (4)	Zn2—N4	2.092 (7)
P1—O3	1.583 (4)	Zn2—N3X^ii^	2.107 (7)
P1—C11	1.818 (5)	Zn2—N3X	2.107 (7)
O3—H3C	0.8597	Zn2—N4X^ii^	2.099 (7)
O4—C23	1.251 (8)	Zn2—N4X	2.099 (7)
O5—C23	1.258 (8)	O1W—H1WA	0.8666
N1—H1	0.9800	O1W—H1WB	0.8636
N1—C1	1.454 (8)	N3—H3	0.9800
N1—C5	1.493 (8)	N3—C6	1.471 (9)
N2—H2	0.9800	N3—C10	1.447 (8)
N2—C3	1.487 (8)	N4—H4	0.9800
N2—C4	1.459 (8)	N4—C8	1.443 (8)
C1—H1A	0.9700	N4—C9	1.461 (9)
C1—H1B	0.9700	C6—H6A	0.9696
C1—C2	1.531 (9)	C6—H6B	0.9700
C2—H2A	0.9700	C6—C7	1.522 (11)
C2—H2B	0.9700	C7—H7A	0.9697
C2—C3	1.490 (9)	C7—H7B	0.9701
C3—H3A	0.9700	C7—C8	1.514 (11)
C3—H3B	0.9700	C8—H8A	0.9699
C4—H4A	0.9700	C8—H8B	0.9701
C4—H4B	0.9700	C9—H9A	0.9700
C4—C5^i^	1.522 (9)	C9—H9B	0.9700
C5—H5A	0.9700	C9—C10^ii^	1.48 (2)
C5—H5B	0.9700	C10—H10A	0.9697
C11—C12	1.384 (8)	C10—H10B	0.9702
C11—C16	1.389 (7)	N3X—H3X	0.9800
C12—H12	0.9300	N3X—C6X	1.475 (8)
C12—C13	1.382 (8)	N3X—C10X	1.460 (10)
C13—H13	0.9300	N4X—H4X	0.9800
C13—C14	1.394 (8)	N4X—C8X	1.462 (9)
C14—C15	1.388 (8)	N4X—C9X	1.473 (8)
C14—C17	1.496 (7)	C6X—H6XA	0.9702
C15—H15	0.9300	C6X—H6XB	0.9699
C15—C16	1.379 (8)	C6X—C7X	1.544 (11)
C16—H16	0.9300	C7X—H7XA	0.9700
C17—C18	1.401 (8)	C7X—H7XB	0.9700
C17—C22	1.381 (8)	C7X—C8X	1.527 (11)
C18—H18	0.9300	C8X—H8XA	0.9700
C18—C19	1.382 (8)	C8X—H8XB	0.9694
C19—H19	0.9300	C9X—H9XA	0.9700
C19—C20	1.363 (8)	C9X—H9XB	0.9700
C20—C21	1.383 (8)	C9X—C10X^ii^	1.58 (3)
C20—C23	1.513 (8)	C10X—H10C	0.9699
C21—H21	0.9300	C10X—H10D	0.9699
			
O1^i^—Zn1—O1	180.0	N3^ii^—Zn2—N3	180.0
N1^i^—Zn1—O1	88.15 (16)	N3^ii^—Zn2—N3X^ii^	21.5 (3)
N1—Zn1—O1	91.85 (16)	N3—Zn2—N3X^ii^	158.5 (3)
N1^i^—Zn1—O1^i^	91.85 (16)	N4^ii^—Zn2—O1W	83.7 (3)
N1—Zn1—O1^i^	88.15 (16)	N4—Zn2—O1W^ii^	83.7 (3)
N1^i^—Zn1—N1	180.0	N4—Zn2—O1W	96.3 (3)
N1^i^—Zn1—N2	85.27 (19)	N4^ii^—Zn2—O1W^ii^	96.3 (3)
N1—Zn1—N2^i^	85.27 (19)	N4—Zn2—N3	96.8 (4)
N1^i^—Zn1—N2^i^	94.73 (18)	N4^ii^—Zn2—N3^ii^	96.8 (4)
N1—Zn1—N2	94.73 (18)	N4—Zn2—N3^ii^	83.2 (4)
N2—Zn1—O1^i^	90.00 (15)	N4^ii^—Zn2—N3	83.2 (4)
N2—Zn1—O1	90.00 (15)	N4^ii^—Zn2—N4	180.0
N2^i^—Zn1—O1	90.00 (15)	N4—Zn2—N3X^ii^	62.3 (4)
N2^i^—Zn1—O1^i^	90.00 (15)	N4^ii^—Zn2—N3X^ii^	117.7 (4)
N2^i^—Zn1—N2	180.0 (2)	N4—Zn2—N4X^ii^	155.7 (3)
O1—P1—O2	116.2 (2)	N4^ii^—Zn2—N4X^ii^	24.3 (3)
O1—P1—O3	110.1 (2)	N3X—Zn2—O1W	90.3 (3)
O1—P1—C11	109.0 (2)	N3X^ii^—Zn2—O1W	89.7 (3)
O2—P1—O3	110.9 (2)	N3X^ii^—Zn2—N3X	180.00 (19)
O2—P1—C11	107.0 (2)	N4X—Zn2—O1W	88.2 (3)
O3—P1—C11	102.6 (2)	N4X^ii^—Zn2—O1W	91.8 (3)
P1—O1—Zn1	135.3 (2)	N4X^ii^—Zn2—N3X	85.0 (4)
P1—O3—H3C	104.2	N4X—Zn2—N3X	95.0 (4)
Zn1—N1—H1	107.3	Zn2—O1W—H1WA	102.4
C1—N1—Zn1	115.2 (4)	Zn2—O1W—H1WB	107.2
C1—N1—H1	107.3	H1WA—O1W—H1WB	88.9
C1—N1—C5	114.3 (5)	Zn2—N3—H3	106.9
C5—N1—Zn1	105.1 (4)	C6—N3—Zn2	117.6 (8)
C5—N1—H1	107.3	C6—N3—H3	106.9
Zn1—N2—H2	108.4	C10—N3—Zn2	107.4 (8)
C3—N2—Zn1	112.3 (4)	C10—N3—H3	106.9
C3—N2—H2	108.4	C10—N3—C6	110.5 (11)
C4—N2—Zn1	104.6 (4)	Zn2—N4—H4	106.5
C4—N2—H2	108.4	C8—N4—Zn2	115.6 (9)
C4—N2—C3	114.4 (5)	C8—N4—H4	106.5
N1—C1—H1A	109.2	C8—N4—C9	116.2 (12)
N1—C1—H1B	109.2	C9—N4—Zn2	105.0 (9)
N1—C1—C2	112.1 (5)	C9—N4—H4	106.5
H1A—C1—H1B	107.9	N3—C6—H6A	109.0
C2—C1—H1A	109.2	N3—C6—H6B	109.5
C2—C1—H1B	109.2	N3—C6—C7	112.2 (12)
C1—C2—H2A	108.0	H6A—C6—H6B	107.7
C1—C2—H2B	108.0	C7—C6—H6A	107.4
H2A—C2—H2B	107.3	C7—C6—H6B	110.9
C3—C2—C1	117.1 (5)	C6—C7—H7A	103.5
C3—C2—H2A	108.0	C6—C7—H7B	104.6
C3—C2—H2B	108.0	H7A—C7—H7B	109.5
N2—C3—C2	111.6 (5)	C8—C7—C6	130.5 (14)
N2—C3—H3A	109.3	C8—C7—H7A	103.1
N2—C3—H3B	109.3	C8—C7—H7B	104.7
C2—C3—H3A	109.3	N4—C8—C7	113.1 (12)
C2—C3—H3B	109.3	N4—C8—H8A	109.8
H3A—C3—H3B	108.0	N4—C8—H8B	108.4
N2—C4—H4A	109.6	C7—C8—H8A	111.0
N2—C4—H4B	109.6	C7—C8—H8B	106.8
N2—C4—C5^i^	110.3 (5)	H8A—C8—H8B	107.6
H4A—C4—H4B	108.1	N4—C9—H9A	109.0
C5^i^—C4—H4A	109.6	N4—C9—H9B	109.0
C5^i^—C4—H4B	109.6	N4—C9—C10^ii^	113.0 (14)
N1—C5—C4^i^	109.3 (5)	H9A—C9—H9B	107.8
N1—C5—H5A	109.8	C10^ii^—C9—H9A	109.0
N1—C5—H5B	109.8	C10^ii^—C9—H9B	109.0
C4^i^—C5—H5A	109.8	N3—C10—C9^ii^	106.5 (14)
C4^i^—C5—H5B	109.8	N3—C10—H10A	110.4
H5A—C5—H5B	108.3	N3—C10—H10B	110.5
C12—C11—P1	124.4 (4)	C9^ii^—C10—H10A	109.7
C12—C11—C16	118.0 (5)	C9^ii^—C10—H10B	110.2
C16—C11—P1	117.6 (4)	H10A—C10—H10B	109.5
C11—C12—H12	119.6	Zn2—N3X—H3X	106.4
C13—C12—C11	120.9 (5)	C6X—N3X—Zn2	112.4 (8)
C13—C12—H12	119.6	C6X—N3X—H3X	106.4
C12—C13—H13	119.5	C10X—N3X—Zn2	108.7 (9)
C12—C13—C14	121.0 (6)	C10X—N3X—H3X	106.4
C14—C13—H13	119.5	C10X—N3X—C6X	115.9 (12)
C13—C14—C17	121.6 (5)	Zn2—N4X—H4X	108.9
C15—C14—C13	118.0 (5)	C8X—N4X—Zn2	113.3 (8)
C15—C14—C17	120.4 (5)	C8X—N4X—H4X	108.9
C14—C15—H15	119.7	C8X—N4X—C9X	112.4 (10)
C16—C15—C14	120.6 (5)	C9X—N4X—Zn2	104.2 (8)
C16—C15—H15	119.7	C9X—N4X—H4X	108.9
C11—C16—H16	119.3	N3X—C6X—H6XA	108.3
C15—C16—C11	121.5 (6)	N3X—C6X—H6XB	108.4
C15—C16—H16	119.3	N3X—C6X—C7X	116.4 (13)
C18—C17—C14	120.9 (5)	H6XA—C6X—H6XB	107.4
C22—C17—C14	121.5 (6)	C7X—C6X—H6XA	107.7
C22—C17—C18	117.6 (5)	C7X—C6X—H6XB	108.3
C17—C18—H18	119.8	C6X—C7X—H7XA	109.6
C19—C18—C17	120.4 (5)	C6X—C7X—H7XB	109.6
C19—C18—H18	119.8	H7XA—C7X—H7XB	108.1
C18—C19—H19	119.1	C8X—C7X—C6X	110.4 (13)
C20—C19—C18	121.8 (6)	C8X—C7X—H7XA	109.6
C20—C19—H19	119.1	C8X—C7X—H7XB	109.6
C19—C20—C21	118.1 (5)	N4X—C8X—C7X	112.2 (12)
C19—C20—C23	121.5 (6)	N4X—C8X—H8XA	108.6
C21—C20—C23	120.3 (6)	N4X—C8X—H8XB	110.0
C20—C21—H21	119.5	C7X—C8X—H8XA	108.6
C20—C21—C22	121.1 (6)	C7X—C8X—H8XB	109.4
C22—C21—H21	119.5	H8XA—C8X—H8XB	107.9
C17—C22—C21	121.0 (6)	N4X—C9X—H9XA	109.3
C17—C22—H22	119.5	N4X—C9X—H9XB	109.4
C21—C22—H22	119.5	N4X—C9X—C10X^ii^	111.5 (14)
O4—C23—O5	125.7 (6)	H9XA—C9X—H9XB	108.0
O4—C23—C20	117.1 (6)	C10X^ii^—C9X—H9XA	108.9
O5—C23—C20	117.3 (6)	C10X^ii^—C9X—H9XB	109.6
O1W^ii^—Zn2—O1W	180.0	N3X—C10X—H10C	110.9
N3—Zn2—O1W^ii^	92.6 (3)	N3X—C10X—H10D	110.4
N3^ii^—Zn2—O1W^ii^	87.4 (3)	C9X^ii^—C10X—H10C	110.9
N3^ii^—Zn2—O1W	92.6 (3)	C9X^ii^—C10X—H10D	110.2
N3—Zn2—O1W	87.4 (3)	H10C—C10X—H10D	108.8
			
Zn1—N1—C1—C2	54.5 (6)	C17—C14—C15—C16	−179.5 (5)
Zn1—N1—C5—C4^i^	−40.5 (5)	C17—C18—C19—C20	2.4 (9)
Zn1—N2—C3—C2	−58.8 (5)	C18—C17—C22—C21	−1.9 (9)
Zn1—N2—C4—C5^i^	41.9 (5)	C18—C19—C20—C21	−2.6 (9)
P1—C11—C12—C13	179.7 (4)	C18—C19—C20—C23	174.7 (5)
P1—C11—C16—C15	179.5 (4)	C19—C20—C21—C22	0.5 (9)
O1—P1—C11—C12	−73.1 (5)	C19—C20—C23—O4	13.2 (8)
O1—P1—C11—C16	107.0 (4)	C19—C20—C23—O5	−167.7 (6)
O2—P1—O1—Zn1	−6.4 (4)	C20—C21—C22—C17	1.7 (9)
O2—P1—C11—C12	160.4 (4)	C21—C20—C23—O4	−169.6 (6)
O2—P1—C11—C16	−19.5 (5)	C21—C20—C23—O5	9.5 (8)
O3—P1—O1—Zn1	120.7 (3)	C22—C17—C18—C19	−0.1 (8)
O3—P1—C11—C12	43.6 (5)	C23—C20—C21—C22	−176.8 (5)
O3—P1—C11—C16	−136.3 (4)	Zn2—N3—C6—C7	41.4 (15)
N1—C1—C2—C3	−72.0 (7)	Zn2—N3—C10—C9^ii^	−42.7 (14)
C1—N1—C5—C4^i^	−167.7 (5)	Zn2—N4—C8—C7	−48.3 (14)
C1—C2—C3—N2	74.9 (7)	Zn2—N4—C9—C10^ii^	39.5 (19)
C3—N2—C4—C5^i^	165.2 (5)	Zn2—N3X—C6X—C7X	56.2 (14)
C4—N2—C3—C2	−177.8 (5)	Zn2—N3X—C10X—C9X^ii^	−38.2 (17)
C5—N1—C1—C2	176.4 (5)	Zn2—N4X—C8X—C7X	−63.1 (13)
C11—P1—O1—Zn1	−127.4 (3)	Zn2—N4X—C9X—C10X^ii^	43.1 (13)
C11—C12—C13—C14	0.5 (9)	N3—C6—C7—C8	−53 (2)
C12—C11—C16—C15	−0.4 (8)	C6—N3—C10—C9^ii^	−172.2 (12)
C12—C13—C14—C15	0.2 (8)	C6—C7—C8—N4	58 (2)
C12—C13—C14—C17	178.7 (5)	C8—N4—C9—C10^ii^	168.5 (14)
C13—C14—C15—C16	−1.0 (8)	C9—N4—C8—C7	−172.0 (13)
C13—C14—C17—C18	40.4 (8)	C10—N3—C6—C7	165.1 (12)
C13—C14—C17—C22	−139.4 (6)	N3X—C6X—C7X—C8X	−73.0 (16)
C14—C15—C16—C11	1.1 (9)	C6X—N3X—C10X—C9X^ii^	−166.0 (12)
C14—C17—C18—C19	−179.9 (5)	C6X—C7X—C8X—N4X	75.3 (16)
C14—C17—C22—C21	177.9 (5)	C8X—N4X—C9X—C10X^ii^	166.2 (12)
C15—C14—C17—C18	−141.2 (6)	C9X—N4X—C8X—C7X	179.0 (11)
C15—C14—C17—C22	39.0 (8)	C10X—N3X—C6X—C7X	−177.9 (14)
C16—C11—C12—C13	−0.4 (8)		

Symmetry codes: (i) −*x*+2, −*y*, −*z*+2; (ii) −*x*+2, −*y*, −*z*+1.

### Hydrogen-bond geometry (Å, º)

**Table:**

*D*—H···*A*	*D*—H	H···*A*	*D*···*A*	*D*—H···*A*
N1—H1···O2	0.98	1.98	2.923 (6)	161
N2—H2···O4^iii^	0.98	2.26	3.220 (6)	166
N3—H3···O3	0.98	2.11	3.076 (10)	168
N4—H4···O5^iii^	0.98	1.84	2.815 (11)	178
N3*X*—H3*X*···O3	0.98	2.33	3.239 (11)	155
N4*X*—H4*X*···O5^iii^	0.98	2.19	3.075 (11)	150
O3—H3*C*···O4^iii^	0.86	1.75	2.597 (6)	167
O1*W*—H1*WA*···O2^ii^	0.87	2.08	2.735 (5)	132
O1*W*—H1*WB*···O5^iv^	0.86	1.82	2.668 (6)	169

Symmetry codes: (ii) −*x*+2, −*y*, −*z*+1; (iii) *x*+1, *y*−1, *z*; (iv) −*x*+1, −*y*+1, −*z*+1.
